# Healthy dietary pattern is associated with lower glycemia independently of the genetic risk of type 2 diabetes: a cross-sectional study in Finnish men

**DOI:** 10.1007/s00394-024-03444-5

**Published:** 2024-06-12

**Authors:** Ulla Tolonen, Maria Lankinen, Markku Laakso, Ursula Schwab

**Affiliations:** 1 Institute of Public Health and Clinical Nutrition, University of Eastern Finland, PO Box 1627, Kuopio, 70211 Finland; 2 Institute of Clinical Medicine, Internal Medicine, University of Eastern Finland, Kuopio, Finland; 3 Department of Medicine, Kuopio University Hospital, Kuopio, Finland; 4 Department of Medicine, Endocrinology and Clinical Nutrition, Kuopio University Hospital, Kuopio, Finland

**Keywords:** Diet, Gene, Human, Glucose, Glycemia, Diabetes

## Abstract

**Purpose:**

Hyperglycemia is affected by lifestyle and genetic factors. We investigated if dietary patterns associate with glycemia in individuals with high or low genetic risk for type 2 diabetes (T2D).

**Methods:**

Men (*n* = 1577, 51–81 years) without T2D from the Metabolic Syndrome in Men (METSIM) cohort filled a food-frequency questionnaire and participated in a 2-hour oral glucose tolerance test. Polygenetic risk score (PRS) including 76 genetic variants was used to stratify participants into low or high T2D risk groups. We established two data-driven dietary patterns, termed healthy and unhealthy, and investigated their association with plasma glucose concentrations and hyperglycemia risk.

**Results:**

Healthy dietary pattern was associated with lower fasting and 2-hour plasma glucose, glucose area under the curve, and better insulin sensitivity (Matsuda insulin sensitivity index) and insulin secretion (disposition index) in unadjusted and adjusted models, whereas the unhealthy pattern was not. No interaction was observed between the patterns and PRS on glycemic measures. Healthy dietary pattern was negatively associated with the risk for hyperglycemia in an adjusted model (OR 0.69, 95% CI 0.51–0.95, in the highest tertile), whereas unhealthy pattern was not (OR 1.08, 95% CI 0.79–1.47, in the highest tertile). No interaction was found between diet and PRS on the risk for hyperglycemia (*p* = 0.69 for healthy diet, *p* = 0.54 for unhealthy diet).

**Conclusion:**

Our findings suggest that healthy diet is associated with lower glucose concentrations and lower risk for hyperglycemia in men with no interaction with the genetic risk.

**Supplementary Information:**

The online version contains supplementary material available at 10.1007/s00394-024-03444-5.

## Introduction

Type 2 diabetes (T2D) is increasing globally and is one of the leading causes of morbidity and mortality in the world [1]. It is preceded by worsening glycemia i.e. prediabetes, which itself is a health hazard [2, 3]. Hyperglycemia (prediabetes or T2D) can be successfully prevented or delayed with lifestyle modification (diet and physical activity) [4, 5]. Genetic factors also worsen glycemia [6–8].

Diet is one of the key elements affecting the risk of T2D and hyperglycemia [9]. Dietary patterns describe a typical diet, with a focus on total diet, accounting all foods and beverages, instead of focusing on single foods or nutrients. Healthy dietary patterns, often characterized by high consumption of vegetables, fruits and berries, fish, vegetable oils, whole-grain products, and low-fat dairy, have been associated with lower plasma glucose concentrations, lower risk for hyperglycemia and lower incidence of T2D [10–19]. Accordingly, unhealthy dietary pattern has been associated with worsening of glycemia and the risk for T2D [13, 18, 20–22].

In addition to dietary causes, T2D has a strong genetic background. Whether the response to diet is similar between those with low and high genetic risk is currently limitedly known. There are studies that have investigated the role of single genetic variants or single foods and nutrients on glycemia. E.g. the relationship of dietary fats and carbohydrates on T2D risk may be dependent on glucose-dependent insulinotropic polypeptide receptor genotype [23], and the effect of dietary fibre and whole-grain on glycemia may depend on genetic variants, especially transcription factor-7-like 2 variant [24–27]. Benefits of fruit intake may be modified by genetic risk score [28]. Meat consumption and sugar-sweetened beverages have been linked to higher fasting glucose and insulin concentrations regardless of the polygenic risk score (PRS) [29, 30]. A meta-analysis modelled that replacement of 5 E% of carbohydrates from refined starch and sugars with polyunsaturated fatty acids lowered the risk for T2D without interaction with the PRS [31].

Previous studies investigating the association of glycemic effects of dietary patterns and genetic risk scores are inconclusive. In the Malmö Diet and Cancer Cohort study, the dietary choices were associated with T2D risk independently of the genetic risk score [32, 33]. Similarly, the EPIC-InterAct consortium found no significant interaction between diet and the PRS on T2D risk [34, 35]. A meta-analysis concluded that healthy diet is associated with decreased fasting glucose and insulin concentrations without interaction with fasting glucose and insulin-related loci [36]. However, some previous studies have indicated that hyperglycemic or protective effects of diet are influenced by the PRS. Health Professionals Follow-up Study found a significant interaction between the high genetic risk and unhealthy, western diet on T2D risk [37]. Similar results have been found in Korean population [38]. A large study from the UK Biobank showed that the protective effect of diet is modified by PRS; especially those in the highest risk benefited from healthy diet [39].

The aim of this study was to establish data-driven dietary patterns and investigate their effect on plasma glucose concentration and the risk for hyperglycemia in middle-aged to elderly Finnish men without prior T2D taking also account of the genetic risk for T2D.

## Methods

### Study participants

**Fig. 1:**
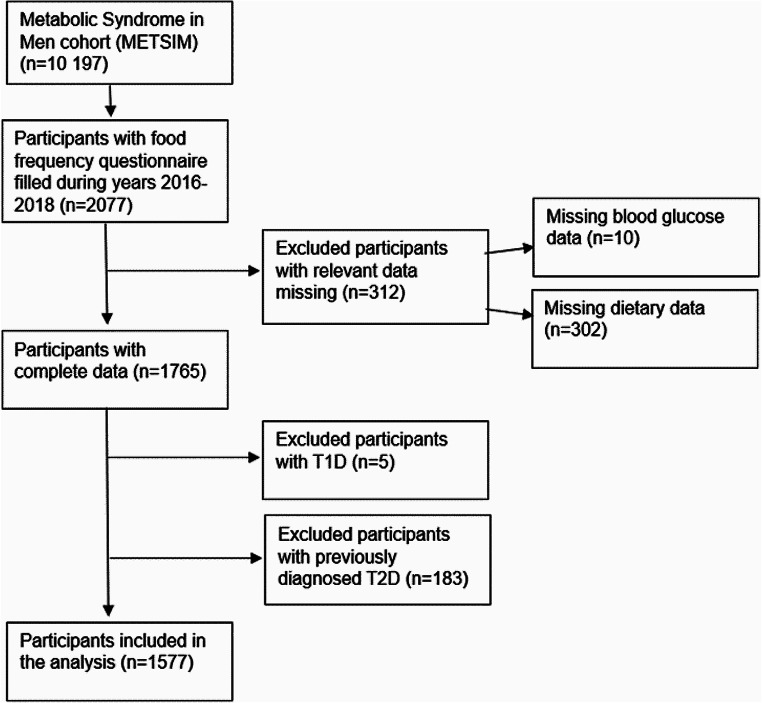
Participant flow

Study participants (*n* = 1577) were Finnish men, aged 51–81 years, attending to the Metabolic Syndrome in Men (METSIM) cohort [40]. A total of 2077 men had a 2-hour oral glucose tolerance test (OGTT), and they filled a food frequency questionnaire (FFQ) during 2016–2018 (Fig. 1). Exclusion criteria were previously diagnosed type 1 or type 2 diabetes, missing relevant dietary data or failed OGTT measurements.

### Clinical assessment

Anthropometric measurements, OGTT procedure and laboratory methods used in this study have been previously described [41]. We defined hyperglycemia as either having prediabetes (fasting plasma glucose (FPG) 5.6–6.9 mmol/l, 2-hour plasma glucose (2-h PG) 7.8–11.0 mmol/l, or HbA1_C_ 5.7–6.4%) or diabetes (FPG ≥ 7.0 mmol/l, 2-h PG ≥ 11.1 mmol/l), or HbA1C ≥ 6.5% (≥ 48 mmol/mol)), according to American Diabetes Association diagnostic criteria [42]. For insulin sensitivity and β-cell function assessment, we calculated Matsuda insulin sensitivity index (Matsuda ISI) and disposition index (DI) as described earlier [41]. Leisure time exercise was divided into two groups, physically active, meaning physical exercise regularly at least 30 min week, and physically inactive with little or no leisure time exercise or physical exercise in context of other hobbies or physical exercise occasionally. For current smoking status we included those who smoke cigarettes, cigars and/or pipes.

### Assessment of diet

We assessed dietary habits using a self-administered qualitative food frequency questionnaire (Online Resource 1, Supplementary Information 1). The questionnaire is a slightly modified version from the FFQ used as a part of Finrisk 2007 National Health Survey basic questionnaire [43]. Consumption frequency of 40 foods were asked using an 8-scale question with reply choices: consumption less than once per month or never, 1–3 times per month, once per week, 2–4 times per week, 5–6 times per week, once per day, 2–3 times per day, or over 4 times per day. Bread consumption was asked for three different bread types using an 8-scale reply choices: less than 1 slice per day or none, 1 slice per week, 2–4 slices per week, 5–6 slices per week, 1 slice per day, 2–3 slices per day, 4–5 slices per day, or 6 or more slices per day. The filling of the questionnaire was instructed by a registered nurse or a clinical nutritionist, and the participants had the possibility to ask for help in filling the questionnaire while attending a study visit.

### Genotyping and genetic risk groups

For genotyping, we used either HumanOmniExpress BeadChip-12v1 (Illumina, San Diego, CA, USA; 733,202 markers) or HumanExome-12v1.1 Beadchip (Illumina, 247,870 markers), as described earlier by the METSIM study group [44]. We calculated PRS based on the 76 genetic variants associated with T2D risk up until 2016 [45, 46]. In the analyses including PRS, we compared low and high risk groups (*n* = 736 and *n* = 699, respectively).

### Statistical analysis

We used SPSS (version 27, IBM Corp., Armonk, NY, USA) in the statistical analysis, and R (version 4.2.2; R Foundation for Statistical Computing, Vienna, Austria) for creating figures. Outcome measurements were concentrations of FPG and 2-h PG, glucose area under the curve (AUC), Matsuda ISI, disposition index, and incidence hyperglycemia.

We used principal components analysis (PCA) to suppress the total of 43 dietary variables into fewer and to create dietary patterns. After assessing the applicability of PCA with Kaiser-Meyer-Olkin Measure of Sampling Adequacy (KMO) and Bartlett’s test of sphericity, with the KMO value of 0.791 and Bartlett’s test of *p* < 0.001, we used PCA with orthogonal (varimax) rotation, with small coefficients with absolute values below 0.3 suppressed. Using Eigenvalue above 1 and PCA scree plot to determine the fixed number of factors, we further extracted 2 dietary components, explaining 17.5% of total cumulative variance in food consumption (Online Resource 2, Supplementary Table 1). We classified the patterns as “healthy” and “unhealthy” (explaining 7.5% and 10% of the variance in food consumption, respectively). Each participant received a value for both dietary patterns.

We then tested how these two dietary components are associated with the glucose variables using linear regression. We performed the statistical analyses with an unadjusted model and adjusted models including body mass index (BMI), age, leisure time exercise (in two categories), current smoking, and total alcohol consumption (g/week). To assess whether the response to dietary pattern is affected by the genetic risk of T2D, we also stratified the participants into the low and high PRS groups. Interaction between the diet and PRS was analysed using a two-way between groups ANOVA.

We used Kolmogorov-Smirnov test to assess the normality. Glucose variables were logarithmically transformed for linear regression and ANOVA analyses. We used untransformed variables for ANOVA to calculate the effect sizes to retain clinically explicable values. We performed a binary logistic regression to assess how the consumption frequency of both healthy or unhealthy foods affect the risk of hyperglycemia (prediabetes or T2D diagnosed at the study visit). For this, we created the tertiles of both healthy and unhealthy food consumption patterns based on subject’s factor scores and used the lowest consumption tertile as the reference category. In addition to unadjusted model including food consumption pattern only, we used models including food consumption pattern, age, BMI, leisure time exercise, current smoking status, and total alcohol consumption. We repeated the analyses stratified with the PRS and looked at the dietary interaction between PRS and dietary pattern. P-values < 0.05 were considered statistically significant for all analyses.

## Results

### Characteristics of the participants

A total of 26.6% (*n* = 420) of participants were normoglycemic, and 73.4% (*n* = 1157) hyperglycemic (of which 898 participants with isolated impaired fasting glucose (IFG), 21 with isolated impaired glucose tolerance (IGT), 157 with both, and 81 had a new diabetes at study visit). Study participants were middle-aged to elderly, mean age was 63.8 years (SD 6.2) and mean BMI 27.2 kg/m^2^ (SD 3.7) (Table 1). The tertiles of dietary pattern for both healthy and unhealthy differed in age, weight, BMI, waist-hip-ratio, leisure time exercise and total alcohol consumption.

**Table 1:** Characteristics of the study population

Characteristic	All (*n* = 1577)	Healthy pattern lowest tertile (*n* = 526)	Healthy pattern middle tertile (*n* = 525)	Healthy pattern highest tertile (*n* = 526)	*p*	Unhealthy pattern lowest tertile (*n* = 525)	Unhealthy pattern middle tertile (*n* = 526)	Unhealthy pattern highest tertile (*n* = 526)	*p*	Low PRS (*n* = 736)	High PRS (*n* = 699)	*p*
Age, years	63.8 ± 6.2	63.3 ± 6.1	63.8 ± 6.1	64.3 ± 6.5	0.020^a^	65.2 ± 5.8	63.8 ± 6.0	62.4 ± 6.5	< 0.001^a^	64.3 ± 6.0	63.9 ± 6.4	0.237^b^
Height, cm	176.3 ± 6.2	176.2 ± 6.3	176.2 ± 6.1	176.5 ± 6.2	0.776^a^	176.0 ± 6.0	176.4 ± 6.2	176.5 ± 6.4	0.312^a^	176.0 ± 6.2	176.5 ± 6.2	0.097^b^
Weight, kg	84.7 ± 12.7	86.1 ± 13.0	84.2 ± 11.8	83.8 ± 13.1	0.002^a^	83.5 ± 12.5	84.9 ± 11.4	85.7 ± 13.6	0.004^a^	85.5 ± 13.2	84.0 ± 11.5	0.122^b^
Body mass index, kg/m^2^	27.2 ± 3.7	27.7 ± 3.8	27.1 ± 3.4	26.9 ± 3.8	< 0.001^a^	26.9 ± 3.6	27.3 ± 3.4	27.5 ± 4.0	0.035^a^	27.6 ± 3.9	26.9 ± 3.2	0.020^b^
Waist-hip-ratio	0.99 ± 0.06	0.99 ± 0.06	0.98 ± 0.06	0.97 ± 0.06	< 0.001^a^	0.98 ± 0.06	0.98 ± 0.06	0.99 ± 0.06	0.031^a^	0.99 ± 0.06	0.98 ± 0.06	< 0.001^b^
Total cholesterol, mmol/l	5.1 ± 1.0	5.1 ± 1.0	5.1 ± 1.0	5.0 ± 1.0	0.338^c^	5.0 ± 1.0	5.2 ± 1.0	5.1 ± 1.0	0.008^c^	5.1 ± 1.0	5.1 ± 1.0	0.557^d^
LDL cholesterol, mmol/l	3.1 ± 0.9	3.1 ± 0.9	3.1 ± 0.9	3.1 ± 0.9	0.659^a^	3.0 ± 0.9	3.2 ± 0.9	3.1 ± 0.9	0.006^a^	3.1 ± 0.9	3.1 ± 0.9	0.811^b^
HDL cholesterol, mmol/l	1.5 ± 0.4	1.4 ± 0.4	1.5 ± 0.4	1.5 ± 0.4	< 0.001^a^	1.5 ± 0.4	1.5 ± 0.4	1.5 ± 0.4	0.561^a^	1.4 ± 0.4	1.5 ± 0.4	0.128^b^
Triglycerides, mmol/l	1.3 ± 0.7	1.4 ± 0.7	1.3 ± 0.9	1.2 ± 0.5	< 0.001^a^	1.3 ± 0.6	1.3 ± 0.8	1.3 ± 0.7	0.109^a^	1.3 ± 0.6	1.3 ± 0.8	0.936^b^
Systolic blood pressure, mmHg	133 ± 15	134 ± 15	134 ± 16	132 ± 15	0.268^a^	134 ± 16	134 ± 15	132 ± 16	0.019^a^	134 ± 16	133 ± 15	0.529^b^
Diastolic blood pressure, mmHg	83 ± 9	84 ± 9	83 ± 9	82 ± 9	0.001^a^	83 ± 9	83 ± 9	83.4 ± 9	0.619^a^	83 ± 9	83 ± 9	0.555^b^
Alcohol consumption^f^, g/week	73.9 ± 102.2	85.0 ± 112.8	77.6 ± 103.2	58.8 ± 86.9	0.045^a^	62.1 ± 98.8	75.8 ± 100.7	83.8 ± 106.0	< 0.001^a^	73.7 ± 99.0	73.5 ± 104.5	0.915^b^
Smoking, current smokers^g^, %	7.8	12.7	7.2	3.3	< 0.001^e^	5.9	7.9	9.6	0.113^e^	8.8	6.3	0.093^e^
Leisure time exercise level, physically active^h^, %	77.5	67.7	78.6	86.3	< 0.001^e^	82.1	77.3	72.9	0.004^e^	75.6	79.5	0.100^e^

Values are mean values with standard deviations unless stated otherwise. P-values < 0.05 are considered statistically significant. P-values according to: ^a^ Kruskal-Wallis test, ^b^ Mann-Whitney U test, ^c^ one-way between groups ANOVA, ^d^ Independent samples t-test, ^e^ Chi-square test for independence. ^f^*n* = 1360, ^g^*n* = 1373, ^h^*n* = 1375. *PRS* polygenic risk score

Participants in the highest healthy dietary pattern tertile were younger, leaner, exercised more and consumed less alcohol than participants in the lower tertiles. The opposite was true for unhealthy dietary pattern. In the healthy pattern, participants in dietary pattern tertiles also differed for concentrations of triglycerides and HDL cholesterol, diastolic blood pressure, and smoking. For unhealthy pattern, tertile differences were significant for a total and LDL cholesterol concentrations, and systolic blood pressure. Low (*n* = 736) and high (*n* = 699) genetic risk groups had statistically significant differences in BMI and waist-to-hip ratio.

### Associations between dietary patterns and plasma glucose concentrations

The healthy dietary pattern was associated with lower concentrations of FPG (β=-0.053, *p* = 0.044), 2-h PG (β=-0.100, *p* < 0.001), lower plasma glucose AUC (β=-0.101, *p* < 0.001), higher Matsuda ISI (β = 0.098, *p* < 0.001) and higher DI (β = 0.065, *p* = 0.014) even after adjusting with BMI, age, leisure time exercise, smoking, and alcohol consumption (Table 2). The unhealthy dietary pattern showed no associations after adjustments.

**Table 2:** Association between healthy and unhealthy dietary patterns and plasma glucose concentrations in all participants

		Model 1		Model 2		Model 3	
		β	*p*	β	*p*	β	*p*
Healthy dietary pattern^1^							
	Fasting plasma glucose (mmol/l)	-0.096	< 0.001	-0.068	0.004	-0.053	0.044
	2-hour plasma glucose (mmol/l)	-0.112	< 0.001	-0.086	< 0.001	-0.100	< 0.001
	Plasma glucose area under the curve (mmol/l * min)	-0.138	< 0.001	-0.109	< 0.001	-0.101	< 0.001
	Matsuda insulin sensitivity index (mg/dl, mU/l)	0.145	< 0.001	0.099	< 0.001	0.098	< 0.001
	Disposition index	0.095	< 0.001	0.068	0.005	0.065	0.014
Unhealthy dietary pattern^2^							
	Fasting plasma glucose (mmol/l)	0.046	0.070	0.026	0.283	0.022	0.406
	2-hour plasma glucose (mmol/l)	-0.006	0.807	-0.025	0.300	0.001	0.963
	Plasma glucose area under the curve (mmol/l * min)	0.020	0.424	-0.001	0.975	0.003	0.903
	Matsuda insulin sensitivity index (mg/dl, mU/l)	-0.053	0.036	-0.019	0.361	-0.023	0.326
	Disposition index	-0.036	0.156	-0.016	0.508	-0.026	0.316

Model 1 = unadjusted values, Model 2 = adjusted with BMI, Model 3 = adjusted with BMI, age, leisure time exercise, smoking, and total alcohol consumption. P-values < 0.05 are considered statistically significant

^1^ High in fresh salad, fresh vegetables; fresh or frozen berries; boiled side vegetables; fruits; oil-based salad dressing or oil with vegetables; fish and fish dishes; chicken, turkey and chicken dishes; unsweetened or artificially sweetened yoghurt (including dairy-, oat-, soy- and rice-based products), quark, Nordic sour milk, or skyr (≤ 1% fat); vegetable dishes; whole grain porridges; whole grain pasta or rice; low-fat cheeses (fat ≤ 17%); boiled or mashed potatoes

^2^ High in fried potatoes or French fries; sausage dishes, sausages; hamburgers; pizza; refined pasta or rice; other sweet pastries; sausage cutleries; other candy; savory pies and pastries; savory snacks; ice cream or puddings; French roll, baquette, or other white bread; sweet cookies, biscuits; meat dishes; ready-meals; other cheeses; sweetened yoghurt (including dairy-, oat-, soy- and rice-based products), quark, or Nordic sour milk (> 1% fat); sour cream based salad dressing

After stratification into the low and high PRS groups and adjusting with BMI, age, leisure time exercise, smoking, and alcohol consumption, we still found an association of healthy dietary pattern with lower 2-h PG, lower glucose AUC, and higher Matsuda ISI in low PRS group, and with higher Matsuda ISI and DI in high PRS group (Table 3).

**Table 3:** Association between the healthy and unhealthy dietary patterns and plasma glucose concentrations stratified by the PRS group (low and high)

		Model 1		Model 2		Model 3	
		β	*p*	β	*p*	β	*p*
Low PRS							
Healthy dietary pattern^1^							
	Fasting plasma glucose (mmol/l)	-0.083	0.025	-0.060	0.081	-0.046	0.229
	2-hour plasma glucose (mmol/l)	-0.117	0.002	-0.096	0.006	-0.130	< 0.001
	Plasma glucose area under the curve (mmol/l * min)	-0.144	< 0.001	-0.121	< 0.001	-0.128	< 0.001
	Matsuda insulin sensitivity index (mg/dl, mU/l)	0.140	< 0.001	0.104	< 0.001	0.113	0.001
	Disposition index	0.076	0.038	0.053	0.122	0.059	0.132
Unhealthy dietary pattern^2^							
	Fasting plasma glucose (mmol/l)	0.054	0.145	0.025	0.466	0.018	0.624
	2-hour plasma glucose (mmol/l)	0.030	0.421	0.003	0.930	0.019	0.625
	Plasma glucose area under the curve (mmol/l * min)	0.041	0.266	0.011	0.741	0.012	0.749
	Matsuda insulin sensitivity index (mg/dl, mU/l)	-0.060	0.102	-0.015	0.630	-0.020	0.559
	Disposition index	-0.060	0.104	-0.030	0.383	-0.035	0.354
High PRS							
Healthy dietary pattern^1^							
	Fasting plasma glucose (mmol/l)	-0.111	0.003	-0.077	0.035	-0.054	0.182
	2-hour plasma glucose (mmol/l)	-0.109	0.004	-0.080	0.031	-0.076	0.061
	Plasma glucose area under the curve (mmol/l * min)	-0.130	< 0.001	-0.094	0.010	-0.070	0.078
	Matsuda insulin sensitivity index (mg/dl, mU/l)	0.161	< 0.001	0.099	0.003	0.095	0.009
	Disposition index	0.140	< 0.001	0.110	0.003	0.092	0.024
Unhealthy dietary pattern^2^							
	Fasting plasma glucose (mmol/l)	0.065	0.085	0.052	0.149	0.046	0.248
	2-hour plasma glucose (mmol/l)	-0.037	0.333	-0.048	0.197	-0.022	0.589
	Plasma glucose area under the curve (mmol/l * min)	0.010	0.795	-0.004	0.915	-0.006	0.877
	Matsuda insulin sensitivity index (mg/dl, mU/l)	-0.027	0.481	-0.004	0.912	0.001	0.974
	Disposition index	-0.022	0.568	-0.010	0.778	-0.015	0.715

Model 1 = unadjusted values, Model 2 = adjusted with BMI, Model 3 = adjusted with BMI, age, leisure time exercise, smoking, and total alcohol consumption. P-values < 0.05 are considered statistically significant. *PRS* polygenic risk score

^1^ High in fresh salad, fresh vegetables; fresh or frozen berries; boiled side vegetables; fruits; oil-based salad dressing or oil with vegetables; fish and fish dishes; chicken, turkey and chicken dishes; unsweetened or artificially sweetened yoghurt (including dairy-, oat-, soy- and rice-based products), quark, Nordic sour milk, or skyr (≤ 1% fat); vegetable dishes; whole grain porridges; whole grain pasta or rice; low-fat cheeses (fat ≤ 17%); boiled or mashed potatoes

^2^ High in fried potatoes or French fries; sausage dishes, sausages; hamburgers; pizza; refined pasta or rice; other sweet pastries; sausage cutleries; other candy; savory pies and pastries; savory snacks; ice cream or puddings; French roll, baquette, or other white bread; sweet cookies, biscuits; meat dishes; ready-meals; other cheeses; sweetened yoghurt (including dairy-, oat-, soy- and rice-based products), quark, or Nordic sour milk (> 1% fat); sour cream based salad dressing

**Fig. 2:**
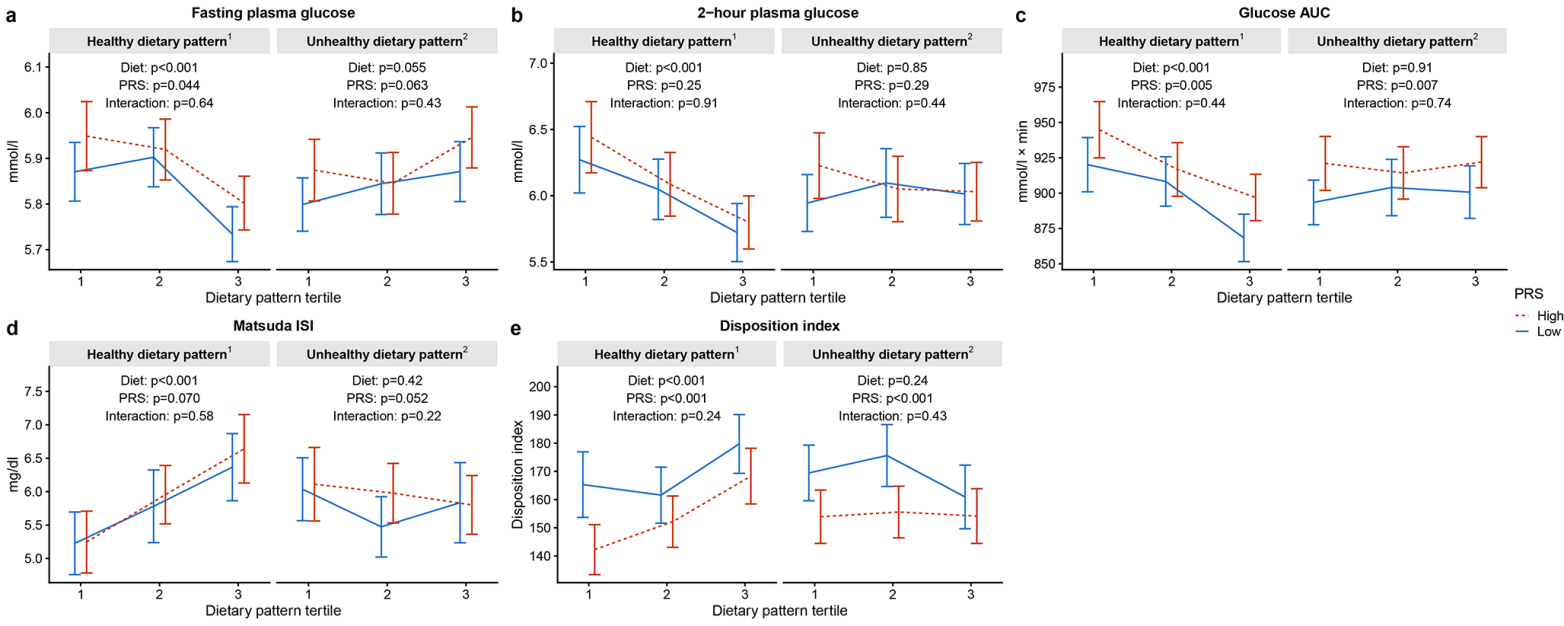
The interaction between dietary patterns (consumption tertiles) and polygenetic risk score (PRS, low and high groups) on glucose variables (means), p-values for interaction from two-way between groups ANOVA. P-values < 0.05 are considered statistically significant. *PRS* polygenic risk score; *Glucose AUC* glucose area under the curve; *Matsuda ISI* Matsuda insulin sensitivity index. ^1^ High in fresh salad, fresh vegetables; fresh or frozen berries; boiled side vegetables; fruits; oil-based salad dressing or oil with vegetables; fish and fish dishes; chicken, turkey and chicken dishes; unsweetened or artificially sweetened yoghurt (including dairy-, oat-, soy- and rice-based products), quark, Nordic sour milk, or skyr (≤ 1% fat); vegetable dishes; whole grain porridges; whole grain pasta or rice; low-fat cheeses (fat ≤ 17%); boiled or mashed potatoes. ^2^ High in fried potatoes or French fries; sausage dishes, sausages; hamburgers; pizza; refined pasta or rice; other sweet pastries; sausage cutleries; other candy; savory pies and pastries; savory snacks; ice cream or puddings; French roll, baquette, or other white bread; sweet cookies, biscuits; meat dishes; ready-meals; other cheeses; sweetened yoghurt (including dairy-, oat-, soy- and rice-based products), quark, or Nordic sour milk (> 1% fat); sour cream based salad dressing

We found no interaction between the diet and PRS on the blood glucose variables on either healthy or unhealthy dietary pattern (Fig. 2). Both low and high PRS groups seem to behave in a similar manner across different tertiles of healthy dietary pattern.

### Risk for hyperglycemia

**Fig. 3:**
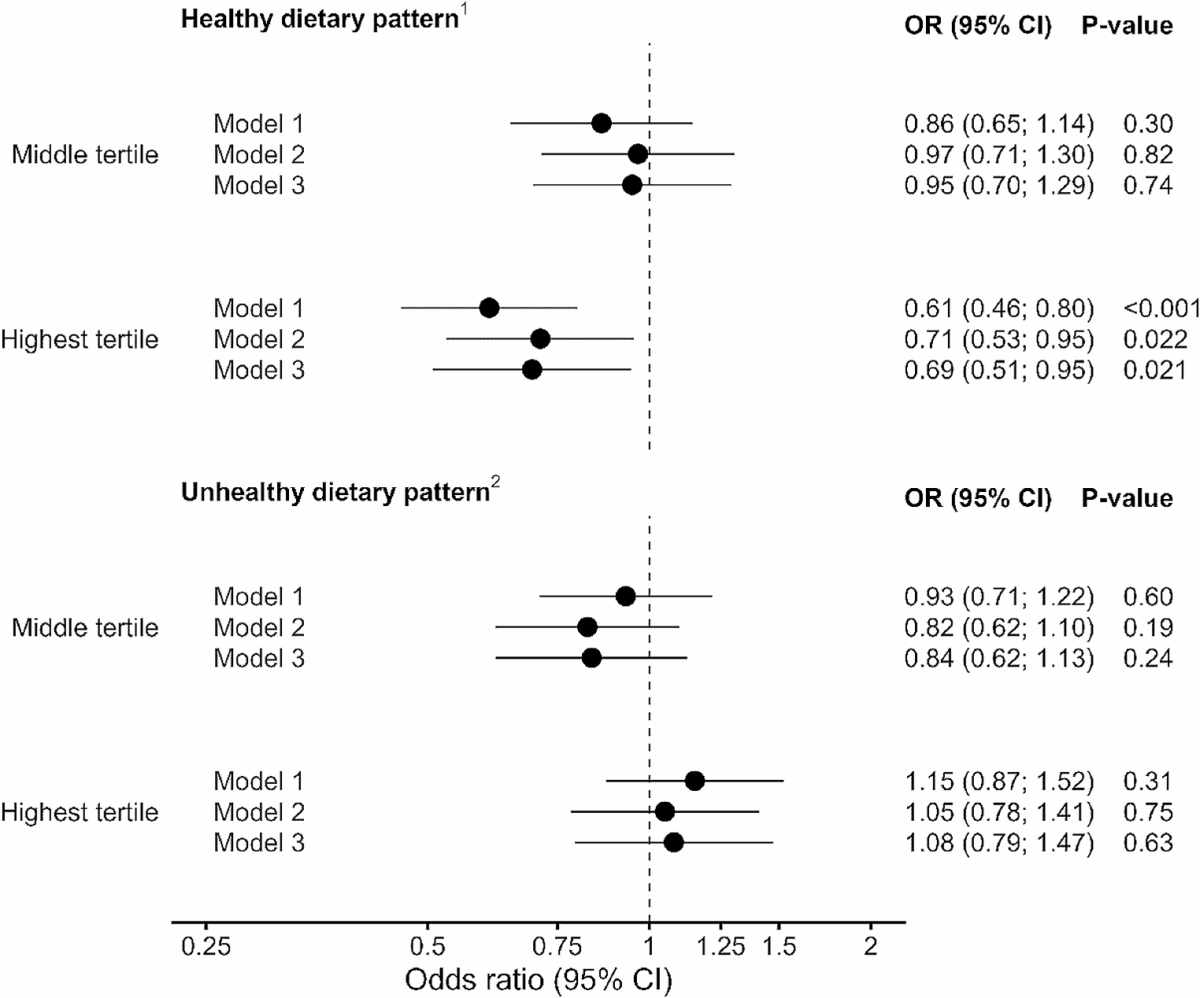
Dietary patterns and the risk of hyperglycemia (= prediabetes or newly diagnosed type 2 diabetes) in all participants. Model 1 = unadjusted values, Model 2 = adjusted with BMI, Model 3 = adjusted with BMI, age, leisure time exercise, smoking, and total alcohol consumption. P-values < 0.05 are considered statistically significant. ^1^ High in fresh salad, fresh vegetables; fresh or frozen berries; boiled side vegetables; fruits; oil-based salad dressing or oil with vegetables; fish and fish dishes; chicken, turkey and chicken dishes; unsweetened or artificially sweetened yoghurt (including dairy-, oat-, soy- and rice-based products), quark, Nordic sour milk, or skyr (≤ 1% fat); vegetable dishes; whole grain porridges; whole grain pasta or rice; low-fat cheeses (fat ≤ 17%); boiled or mashed potatoes. ^2^ High in fried potatoes or French fries; sausage dishes, sausages; hamburgers; pizza; refined pasta or rice; other sweet pastries; sausage cutleries; other candy; savory pies and pastries; savory snacks; ice cream or puddings; French roll, baquette, or other white bread; sweet cookies, biscuits; meat dishes; ready-meals; other cheeses; sweetened yoghurt (including dairy-, oat-, soy- and rice-based products), quark, or Nordic sour milk (> 1% fat); sour cream based salad dressing

Highest tertile of healthy dietary pattern was associated with lower risk for hyperglycemia when all participants were considered (odds ratio (OR) 0.61, 95% CI 0.46–0.80, *p* < 0.001) as compared to the lowest tertile (Fig. 3). The lower risk remained statistically significant after adjusting with BMI, age, leisure time exercise, smoking, and alcohol consumption (OR 0.69, 95% CI 0.51–0.94, *p* = 0.021). For the middle tertile, there was no significant difference in the hyperglycemia risk in either unadjusted or adjusted models. When we compared the highest tertile of unhealthy dietary pattern to the lowest tertile, we saw no significant results in either unadjusted or fully adjusted models.

**Fig. 4:**
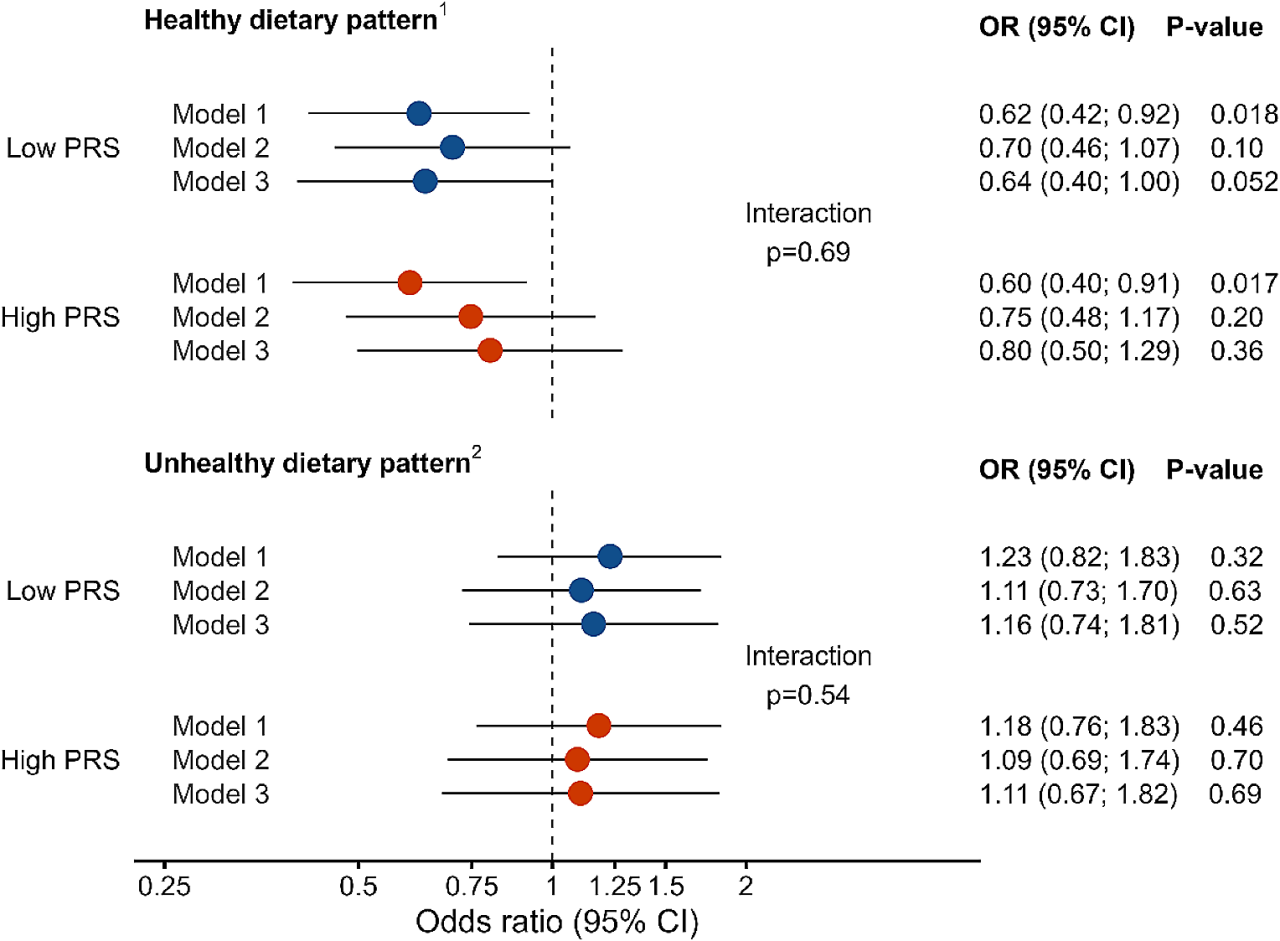
Risk for hyperglycemia in low and high polygenetic risk score (PRS) group in the highest dietary pattern tertiles. Model 1 = unadjusted values, Model 2 = adjusted with BMI, Model 3 = adjusted with BMI, age, leisure time exercise, smoking, and total alcohol consumption. P-values < 0.05 are considered statistically significant. *PRS* polygenic risk score. ^1^ High in fresh salad, fresh vegetables; fresh or frozen berries; boiled side vegetables; fruits; oil-based salad dressing or oil with vegetables; fish and fish dishes; chicken, turkey and chicken dishes; unsweetened or artificially sweetened yoghurt (including dairy-, oat-, soy- and rice-based products), quark, Nordic sour milk, or skyr (≤ 1% fat); vegetable dishes; whole grain porridges; whole grain pasta or rice; low-fat cheeses (fat ≤ 17%); boiled or mashed potatoes. ^2^ High in fried potatoes or French fries; sausage dishes, sausages; hamburgers; pizza; refined pasta or rice; other sweet pastries; sausage cutleries; other candy; savory pies and pastries; savory snacks; ice cream or puddings; French roll, baquette, or other white bread; sweet cookies, biscuits; meat dishes; ready-meals; other cheeses; sweetened yoghurt (including dairy-, oat-, soy- and rice-based products), quark, or Nordic sour milk (> 1% fat); sour cream based salad dressing

After stratification by PRS, we saw that the healthy dietary pattern was associated with lower risk of hyperglycemia in both PRS groups in an unadjusted model (OR 0.62, 95% CI 0.42–0.92, *p* = 0.018, for low GRS, and OR 0.60, 95% CI 0.40–0.91, *p* = 0.017, for high GRS) (Fig. 4). After adjusting with BMI alone, or with BMI, age, leisure time exercise, smoking, and alcohol consumption, the association was no longer significant. Unhealthy diet was not associated with hyperglycemia risk in either PRS group. PRS did not seem to modify the effect of dietary patterns on the hyperglycemia risk (P interaction 0.69 for healthy diet, and P interaction 0.54 for unhealthy diet).

## Discussion

Our cross-sectional study suggests that healthy dietary pattern is associated with lower plasma glucose concentrations and lower risk for hyperglycemia in middle-aged and elderly Finnish men. These results remained statistically significant after adjusting for confounding factors.

When the participants were stratified by their polygenetic risk score, healthy diet was still associated with lower plasma glucose concentrations in both low and high risk groups. The significance remained even after adjusting with BMI for all variables except for FPG in the low PRS group. After adjusting with age and other lifestyle factors (exercise, smoking and alcohol consumption), the healthy dietary pattern was associated with lower 2-h PG, gluc AUC, and higher Matsuda in the low PRS group, and with higher Matsuda and DI in the high PRS group. Both PRS groups seem to benefit from a healthy diet. There was no association between the unhealthy pattern and plasma glucose concentrations.

Healthy dietary pattern was associated with lower risk of hyperglycemia in both low and high genetic risk groups in an unadjusted model. A significant association was, however, lost after the adjustment for BMI. This may imply that the effects of a healthy dietary pattern in the hyperglycemia risk are mediated by BMI. Since adjusting with lifestyle factors beyond diet, such as smoking, exercise, and alcohol consumption, erases the statistical significance, it may be that the benefits of a healthy diet are confounded by an overall healthier lifestyle. The lack of statistical significance after the adjustments can also be due to lack of power when the study population has been divided into smaller groups. Our results suggest that focusing on adding healthy food choices is more essential than avoidance of unhealthy choices.

We found no interaction between the dietary patterns and the PRS in plasma glucose concentrations or in the risk of hyperglycemia. This suggests that the genetic risk does not modify the possible effects of diet.

Similarly to our data-driven pattern, foods such as fruits and berries, vegetables, non-tropical vegetable oils, whole grain products, fish, and low-fat dairy products, load into many established patterns such as Mediterranean Diet Score (MDS), Dietary Approaches to Stop Hypertension (DASH), Healthy Eating Index (HEI), Healthy Nordic Food Index (HNFI) and Baltic Sea Diet Score (BSDS) [47–51]. The two dietary patterns derived with PCA explained 17.5% of the food consumption variance. This level of explanatory value is in line with some similar data-driven dietary pattern studies [16, 37]. Since the principal component analysis aims to distinguish differences in dietary habits, there can be dietary components that are either beneficial or harmful for glucose metabolism that are not shown in the patterns. The dietary patterns identified by principal component analysis only explain a portion of the variance in food consumption. There may be individual dietary components with potential benefits or harmful effects on glucose metabolism that the PCA did not capture.

Our results are in line with previous studies showing beneficial effects of healthy dietary patterns and improved plasma glucose concentration [10], decreased risk for prediabetes [14, 16, 18], and decreased risk for T2D [13–15, 17, 52–55]. There are also some studies showing that unhealthy dietary pattern is associated with inferior plasma glucose concentration [22] and greater risk of prediabetes [18] and T2D [13, 21]. Our study implies similar results, but the results were not statistically significant.

Some previous studies support our findings showing that PRS does not affect the impact of diet on plasma glucose levels [29, 30, 36] or risk of T2D [31–34]. A few studies, however, report that the dietary impact on T2D risk may have interactions with the genetics [23, 28, 35, 37, 38], and that especially those in the highest genetic risk might benefit from healthy diet [39].

The strengths of the study include a rather large study population with oral glucose tolerance test enabling plasma glucose measurements beyond fasting levels, polygenetic risk score, and many variables measured that we were able to use to adjust for confounders.

The limitation of this study is its cross-sectional design. This allows us to make conclusions on associations without the possibility for interpreting causal relationships. Since our study was conducted in middle-aged Finnish men of Caucasian origin, the findings may not be extrapolated to other populations due to e.g. cultural, genetic, and lifestyle differences. Similar studies need to be conducted in females and participants of different ages and ethnicities. Our PRS stratified sample sizes may be inadequate to detect statistical differences across genetic groups. A retrospective food frequency questionnaire is based on the recall of the study participants, and self-reported dietary assessment methods are prone to misreporting [56]. Apart from bread consumption where the consumption was asked with quantitative measures, the qualitative questionnaire captures consumption frequencies of certain foods instead of measuring total food, energy, or nutrient intakes. We have no data on the amounts of foods consumed and there can be aspects of healthy diet that are not captured in this paper due to this limitation. The questionnaire covers a range of typical foods consumed in Finland and can be used to rank participants’ dietary habits. It also measures the usual diet from the past 12 months, which is a rather long period covering seasonal changes in the diet. The questionnaire is, however, a non-exhaustive list of foods consumed, which limits the components included in the dietary patterns. Even with adjustment with covariates such as BMI, age, exercise, smoking, and alcohol consumption, we cannot rule out possible residual confounders that may mediate the results found. We acknowledge the potential risk of multiple testing error, which is always present when more than one endpoint is being tested.

Our results support the existing literature on the protective effect of healthy dietary pattern against hyperglycemia. In addition to reduced risk for T2D, healthy dietary patterns have been shown to reduce risk for cardiovascular diseases, cancer, bone health, mental health, obesity, and premature death [57].

## Conclusion

Our study suggests that a healthy dietary pattern, consisting of foods such as vegetables, fruits and berries, fish, whole-grain products, non-tropical vegetable oils, low-fat yoghurt, poultry, and potatoes, is associated with better plasma glucose concentrations and decreased risk for hyperglycemia.There was no interaction between diet and genetic risk score; it seems that the genetic risk for T2D does not modify the effects of a healthy diet. This highlights the importance of a healthy diet for everyone in maintaining healthy glycemia and preventing T2D. These findings can be used for lifestyle counseling and healthcare planning in diabetes prevention. Further studies with prospective and intervention designs, and with different ethnicities, genders, and ages, are still needed.

## Electronic supplementary material

Below is the link to the electronic supplementary material.

Supplementary Material 1

Supplementary Material 2

## Data Availability

Restrictions apply to the availability of data generated or analyzed during this study to preserve the confidentiality of the participants. The corresponding author will, on request, detail the restrictions and any conditions under which access to some data may be provided.

## Abbreviations

ANOVA: Analysis of variance
BMI: Body mass index
CI: Confidence interval
E%: Percentage of total energy
FFQ: Food frequency questionnaire
FPG: Fasting plasma glucose
HbA1_C_: Hemoglobin A1_C_
HDL: High-density lipoprotein
IFG: Impaired fasting glucose
IGT: Impaired glucose tolerance
KMO: Kaiser-Meyer-Olkin measure of sampling adequacy
LDL: Low-density lipoprotein
Matsuda ISI: Matsuda insulin sensitivity index
METSIM: Metabolic syndrome in men
OGTT: Oral glucose tolerance test
OR: Odds ratio
PCA: Principal component analysis
PG: Plasma glucose
PRS: Polygenetic risk score
SD: Standard deviation
T2D: Type 2 diabetes
