# Supplementary material for: Healthy dietary pattern is associated with lower glycemia independently of the genetic risk of type 2 diabetes: a cross-sectional study in Finnish men
Source: Eur J Nutr. 2024 Jun 12;63(7):2521–31. doi: 10.1007/s00394-024-03444-5 (PMC11490453; doi:10.1007/s00394-024-03444-5)
Supplement: Supplementary file 1 — Supplementary Material 1 [file 394_2024_3444_MOESM1_ESM.docx]

Online Resource 1 to: **Healthy dietary pattern is associated with lower glycemia independently of the genetic risk of type 2 diabetes: a cross-sectional study in Finnish men**

European Journal of Nutrition

Ulla Tolonen^a^, Maria Lankinen^a^, Markku Laakso^b,c^, Ursula Schwab^a,d^

^a^Institute of Public Health and Clinical Nutrition, University of Eastern Finland, Kuopio, Finland

^b^Institute of Clinical Medicine, Internal Medicine, University of Eastern Finland, Kuopio, Finland

^c^Department of Medicine and Clinical Research, Kuopio University Hospital, Kuopio, Finland

^d^Department of Medicine, Endocrinology and Clinical Nutrition, Kuopio University Hospital, Kuopio, Finland

Corresponding author: Ulla Tolonen, Institute of Public Health and Clinical Nutrition, University of Eastern Finland, Po Box 1627, 70211 Kuopio, Finland, e-mail: [ulla.tolonen@uef.fi](mailto:ulla.tolonen@uef.fi)

**Supplementary Information 1** Food frequency questionnaire questions 2 and 3 (the questions from the complete questionnaire that were used in this study)

The questionnaire is a slightly modified version from the FFQ used as a part of Finrisk 2007 National Health Survey basic questionnaire (Finnish Institute for Health and Welfare. The National FINRISK study. Questionnaires. https://thl.fi/documents/189940/4850942/finriski2007questionnaire.pdf/ee4e02c0-1cde-4474-9d4d-9e55697007b5. Accessed 25 Sep 2023).

2. **How often do you usually consume the following foodstuffs (per month, per week, or per day)?** Please consider the past year (12 months). Please provide an answer to each row. Only mark one reply option, the one that best describes the consumption frequency.

Less than once per month or never

1-3 times per month

Once per week

2-4 times per week

5-6 times per week

Once per day

2-3 times per day

Over 4 times per day

[An example is provided on how to mark consumption for porridges 2-4 times per week]

CEREALS:

Buns, bun-based pies

Sweet cookies, biscuits

Other sweet pastries (e.g. cakes, Danish pastry)

Savoury pies and pastries (e.g. Carelian pie)

Pizza

Hamburgers

Refined pasta or rice

Whole grain pasta or rice

Low-fibre porridges (e.g. rice and semolina porridges)

Whole grain porridges (e.g. from oat, rye, or from mixture of oat, rye, barley and wheat)

Breakfast cereals and muesli

DAIRY PRODUCTS

Unsweetened or artificially sweetened yoghurt*, quark, or Nordic sour milk (>1% fat)

Unsweetened or artificially sweetened yoghurt*, quark, Nordic sour milk, or skyr (≤1% fat)

Sweetened yoghurt*, quark, or Nordic sour milk (>1% fat)

Sweetened yoghurt*, quark, Nordic sour milk, or skyr (≤1% fat)

Low-fat cheeses (fat ≤17%, e.g. Edam 17, Oltermanni 17, Polar 10)

Other cheeses (e.g. Edam, Emmental, Aura, Brie)

Ice cream or puddings

*including dairy-, oat-, soy- and rice-based products

POTATO, VEGETABLES

Boiled or mashed potatoes

Fried potatoes or French fries

Vegetable dishes (e.g. soups, casseroles, stews)

Boiled side vegetables

Fresh salad, fresh vegetables

Oil-based salad dressing or oil with vegetables

Sour cream based salad dressing

Non-fatty salad dressing, e.g. fruit juice

FRUITS, BERRIES

Fruits

Fresh or frozen berries

Fruit or berry juices (no added sugar)

FISH

Fish and fish dishes in total

[fish species specific questions were not used in this study]

MEAT, SAUSAGE, EGG

Meat dishes (e.g. roasts, minced meat sauce, steaks)

Chicken, turkey, and chicken dishes

Sausage dishes, sausages

Sausage cutleries (e.g. mettwurst, bologna sausage)

Whole meat cuts (e.g. ham, turkey)

Eggs (boiled, fried, omelets)

OTHER

Chocolate

Other candy

Savory snacks (e.g. chips, popcorn)

Ready-meals

3. **How much do you typically consume different types of bread?** Please consider the past year (12 months). Please provide an answer to each row. Only mark one reply option, the one that best describes the consumption.

Reply choices:

Less than 1 slice per day or none

1 slice per week

2-4 slices per week

5-6 slices per week

1 slice per day

2-3 slices per day

4-5 slices per day

6 or more slices per day

[An example is provided on how to mark consumption for yeast bread once per day]

BREAD

Rye or crisp bread

Yeast bread, graham and whole grain breads including buns and toasts

French roll, baquette, or other white bread
