# Supplementary material for: Healthy dietary pattern is associated with lower glycemia independently of the genetic risk of type 2 diabetes: a cross-sectional study in Finnish men
Source: Eur J Nutr. 2024 Jun 12;63(7):2521–31. doi: 10.1007/s00394-024-03444-5 (PMC11490453; doi:10.1007/s00394-024-03444-5)
Supplement: Supplementary file 2 — Supplementary Material 2 [file 394_2024_3444_MOESM2_ESM.docx]

Online Resource 2 to: **Healthy dietary pattern is associated with lower glycemia independently of the genetic risk of type 2 diabetes: a cross-sectional study in Finnish men**

European Journal of Nutrition

Ulla Tolonen^a^, Maria Lankinen^a^, Markku Laakso^b,c^, Ursula Schwab^a,d^

^a^Institute of Public Health and Clinical Nutrition, University of Eastern Finland, Kuopio, Finland

^b^Institute of Clinical Medicine, Internal Medicine, University of Eastern Finland, Kuopio, Finland

^c^Department of Medicine and Clinical Research, Kuopio University Hospital, Kuopio, Finland

^d^Department of Medicine, Endocrinology and Clinical Nutrition, Kuopio University Hospital, Kuopio, Finland

Corresponding author: Ulla Tolonen, Institute of Public Health and Clinical Nutrition, University of Eastern Finland, Po Box 1627, 70211 Kuopio, Finland, e-mail: [ulla.tolonen@uef.fi](mailto:ulla.tolonen@uef.fi)

**Supplementary Table 1** Factor loadings and dietary patterns derived from principal component analysis for 43 foods

| Food | Healthy dietary pattern (explaining 7.5% of variance in diet) | Unhealthy dietary pattern (explaining 10% of variance in diet) |
| --- | --- | --- |
| Buns, bun-based pies | - | - |
| Sweet cookies, biscuits | - | 0.382 |
| Other sweet pastries (e.g. cakes, Danish pastry) | - | 0.477 |
| Savory pies and pastries (e.g. Carelian pie) | - | 0.440 |
| Pizza | - | 0.493 |
| Hamburger | - | 0.479 |
| Refined pasta or rice | - | 0.468 |
| Whole-grain pasta or rice | 0.364 | - |
| Low-fibre porridges (e.g. rice and semolina porridges) | - | - |
| Whole-grain porridges (e.g. from oat, rye, or from mixture of oat, rye, barley and wheat) | 0.384 | - |
| Rye or crisp bread | - | - |
| Yeast bread, graham and whole grain breads including buns and toasts | - | - |
| French roll, baquette, or other white bread | - | 0.401 |
| Breakfast cereals and muesli | - | - |
| Unsweetened or artificially sweetened yoghurt^1^, quark, or Nordic sour milk with >1% fat | - | - |
| Unsweetened or artificially sweetened yoghurt^1^, quark or Nordic sour milk (≤1% fat) | 0.417 | - |
| Sweetened yoghurt^1^, quark, or Nordic sour milk (>1% fat) | - | 0.325 |
| Sweetened yoghurt^1^, quark, Nordic sour milk, or Skyr (≤1% fat) | - | - |
| Low-fat cheeses (fat ≤17%, e.g. Edam 17, Oltermanni 17, Polar 10) | 0.340 | - |
| Other cheeses (e.g. Edam, Emmental, Aura, Brie) | - | 0.339 |
| Ice cream or puddings | - | 0.409 |
| Boiled or mashed potatoes | 0.309 | - |
| Fried potatoes or French fries | - | 0.561 |
| Vegetable dishes (e.g. soups, casseroles, stews) | 0.407 | - |
| Boiled side vegetables | 0.545 | - |
| Fresh salad, fresh vegetables | 0.628 | - |
| Oil-based salad dressing or oil with vegetables | 0.472 | - |
| Sour cream based salad dressing | - | 0.306 |
| Non-fatty salad dressing (e.g. fruit juice) | - | - |
| Fruits | 0.533 | - |
| Fresh or frozen berries | 0.581 | - |
| Fruit or berry juices (no added sugar) | - | - |
| Fish and fish dishes | 0.451 | - |
| Meat dishes (e.g. roasts, minced meat sauce, steaks) | - | 0.350 |
| Chicken, turkey and chicken dishes | 0.422 | - |
| Sausage dishes, sausages | - | 0.553 |
| Sausage cutleries (e.g. mettwurst, bologna sausage) | - | 0.461 |
| Whole meat cuts (e.g. ham, turkey) | - | - |
| Eggs (boiled, fried, omelets) | - | - |
| Chocolate | - | - |
| Other candy | - | 0.462 |
| Savoury snacks (e.g. chips, popcorn) | - | 0.444 |
| Ready-meals | - | 0.341 |

^1^including dairy-, oat-, soy- and rice-based products
